# Clinical, functional, and immunological profiles across frailty subgroups in maintenance hemodialysis patient

**DOI:** 10.3389/fendo.2026.1799060

**Published:** 2026-05-25

**Authors:** Hongmei Liu, Huanhua Wu, Jiaxin Lin, Huahong Zhou, Xiangjiu Chen, Guobao Hong, Hao Huang, Fanna Liu

**Affiliations:** 1 Department of Nephrology, The Affiliated Shunde Hospital of Jinan University, Foshan, Guangdong, China; 2 Department of Nuclear Medicine, Central People’s Hospital of Zhanjiang, Guangdong Medical University Zhanjiang Central Hospital, Zhanjiang, Guangdong, China; 3 Department of Nephrology, The First Affiliated Hospital of Jinan University, Guangzhou, Guangdong, China

**Keywords:** biomarkers, frailty, immune aging, maintenance hemodialysis, metabolic dysregulation

## Abstract

**Background:**

Frailty is a common syndrome in maintenance hemodialysis (MHD) patients, characterized by diminished physiological reserves and adverse health outcomes. This study explored the associations between clinical, functional, and immunological characteristics across frailty subgroups to improve understanding and guide interventions.

**Methods:**

The single-center observational study included 66 MHD patients categorized into three frailty groups (Normal, Pre-Frailty, Frailty) using Fried FRAIL scale scores. Demographic, clinical, and laboratory data, including physical performance measures, biochemical markers, and immune parameters, were collected. Immune cell subsets and inflammatory cytokines were analyzed via flow cytometry. Subgroup comparisons and correlations between immune and biological age were examined.

**Results:**

Frailty was associated with older age, reduced grip strength, lower physical activity, and higher fall prevalence. Frail patients were older (64.19 ± 14.37 years) with lower grip strength (18.00 [13.60–22.60] kg) compared to Normal patients (26.70 [20.90–30.20] kg, p < 0.001). Elevated ferritin (207.00 [106.50–297.50] μg/L) and HbA1c (6.58 ± 1.75%) levels were observed in the Frailty group. Immune markers, including effector memory CD4^+^ T cells, central memory CD8^+^ T cells, and Th1/Th2 ratios, varied significantly. Immune age correlated strongly with biological age in Normal (r = 0.98) and Pre-Frailty groups (r = 0.96), but weakened in the Frailty group (r = 0.84), suggesting immune dysregulation.

**Conclusions:**

Frailty in MHD patients is linked to distinct clinical, functional, and immunological differences. Immune age may complement traditional frailty measures as a biomarker for assessment and monitoring. A multidimensional approach could improve early detection, risk stratification, and targeted interventions.

## Highlights

- Frailty in maintenance hemodialysis (MHD) patients is characterized by distinct clinical, functional, and immunological differences, emphasizing the need for a comprehensive assessment approach.
- Immune age shows potential as a novel biomarker for frailty evaluation, with its correlation to biological age weakening in advanced frailty, suggesting immune dysregulation.
- Integrating clinical, functional, and immunological evaluations can enhance early frailty detection, risk stratification, and personalized interventions to improve outcomes in this vulnerable population.

## Introduction

Frailty is a clinical syndrome associated with aging, characterized by a progressive decline in physiological reserves, heightened susceptibility to internal and external stressors, and an increased risk of adverse health outcomes. These outcomes include falls, premature mortality, functional decline, hospitalization, and admission to long-term care facilities, all of which significantly impact the quality of life and independence of affected individuals (1–3). Among patients undergoing maintenance hemodialysis (MHD), frailty is both highly prevalent and a pressing concern (4). The physiological strain imposed by MHD further exacerbates frailty, reducing patients’ physiological resilience and amplifying their vulnerability to stressors. These challenges underscore the need for a multidisciplinary approach to improve care and quality of life in this vulnerable population (5).

The interplay between clinical, functional, and immunological characteristics across frailty subgroups in MHD patients remains poorly understood. While studies have examined specific aspects of frailty, such as clinical and functional parameters, the integration of immunological markers has been limited (6, 7). Frailty in MHD patients involves systemic and biological changes, driven by chronic inflammation, immunosenescence, and metabolic dysregulation, which are exacerbated by the physiological demands of hemodialysis (8, 9). Altered immune profiles, including dysregulated T-cell and natural killer (NK) cell activity, are increasingly recognized as contributors to frailty, yet how these immunological changes vary across frailty stratification is largely unexplored. Addressing this gap is essential to understanding the mechanisms underlying frailty and developing targeted, effective interventions for this population.

Recent studies emphasize the importance of assessing frailty in dialysis patients, linking it to adverse outcomes such as hospitalization, mortality, and functional decline (10, 11). Emerging evidence suggests that immune alterations, including changes in T-cell subsets (effector memory CD4+ T cells, central memory CD8+ T cells) and inflammatory markers (Th1/Th2 ratios, inhibitory γδ2+ T cells), contribute to frailty progression by reflecting systemic inflammation and biological aging (12, 13). However, it is unclear whether frailty-associated clinical impairments are directly linked to immune dysfunction or represent independent processes. Furthermore, the variation of these patterns across frailty stages remains poorly understood, offering opportunities for novel diagnostic and therapeutic interventions.

The single-center observational study investigates the associations between clinical, functional, and immunological characteristics across frailty groups in MHD patients to enhance understanding of frailty mechanisms and inform future assessment and intervention strategies.

## Methods

### Participants

The single-center observational study was conducted from January 2023 to March 2024 at the Hemodialysis Centers of Shunde Hospital, Affiliated to Jinan University, and the First Affiliated Hospital of Jinan University. A total of 160 MHD patients were initially screened for eligibility. All participants and their families provided informed consent and voluntarily agreed to participate in the study.

Patients were eligible for inclusion if they were diagnosed with end-stage renal disease (ESRD) based on standard diagnostic criteria, had been undergoing MHD for at least 3 months, were aged 18 years or older, and were receiving regular MHD treatment three times per week. Participants needed to be conscious, capable of understanding and communicating effectively, and willing to cooperate in completing the study questionnaire. Patients were excluded if they had concurrent malignant tumors, liver failure (n = 21); immune system disorders, poisoning, or infections (n = 18); or were unable to complete the survey due to language difficulties, sensory (visual or auditory) impairments, or mental disabilities (n = 10).

Of the 160 patients initially screened, 111 were deemed eligible after applying these criteria. Among them, 45 patients were further excluded due to missing clinical data. A total of 66 patients were included in the final analysis.

### Baseline data collection

Baseline data were collected from all enrolled MHD patients as part of this single-center observational study through structured face-to-face interviews, direct clinical assessments, medical record review, and laboratory analyses. Frailty-related measures, including handgrip strength and FRAIL questionnaire items, were specifically assessed for the purposes of this study rather than being derived solely from routine clinical care. Handgrip strength was measured by trained study personnel using a calibrated electronic hand dynamometer according to a standardized protocol. The preferred testing position was standing, with the participant’s feet placed shoulder-width apart, both arms naturally hanging at the sides, the elbows fully extended, and the palms facing inward while gripping the dynamometer with maximal effort. For participants who were unable to stand independently, grip strength was assessed in a standardized seated position, with the forearm in a neutral position and the wrist extended at 0–30°. Both hands were tested twice, and the highest value obtained across all measurements was used for analysis. Physical activity was assessed through face-to-face interviews as part of the study questionnaire. Participants were classified as physically active if they reported walking at least 6,000 steps per day or accumulating at least 30 minutes of total daily exercise, including all types of physical activity. All data were collected by trained physicians, dialysis nurses, and laboratory personnel according to standardized study procedures to ensure accuracy, consistency, and reliability.

Information included sex, age, dialysis vintage, height, dry weight, body mass index (BMI), handgrip strength, blood pressure, fall history, and physical activity levels. Medical history covered comorbidities such as hypertension, diabetes mellitus, stroke, and coronary artery disease, along with the underlying etiology of ESRD, including chronic glomerulonephritis, diabetes mellitus, hypertension, polycystic kidney disease, obstructive nephropathy, gout-related kidney disease, and autoimmune diseases. Physical activity was defined as engaging in ≥ 6,000 steps per day or accumulating ≥ 30 minutes of daily exercise, including structured and unstructured physical activities.

### Laboratory examination

Pre-dialysis blood samples were collected and analyzed for hemoglobin (Hb), ferritin, creatinine (Cr), uric acid (UA), total calcium (Tca), phosphorus (P), potassium (K), parathyroid hormone (iPTH), albumin (ALB), prealbumin (PA), glucose (GLU), glycated hemoglobin (HbA1c), β2-microglobulin (β2-MG), alkaline phosphatase (ALP), total cholesterol (TC), triglycerides (TG), low-density lipoprotein cholesterol (LDL-C), and high-sensitivity C-reactive protein (CRP). All biochemical analyses were conducted in the hospital’s central laboratory using standardized clinical chemistry methods.

Peripheral blood samples (4 mL) were processed using flow cytometry to analyze immune cell subsets, including T-cell subsets (helper T cells, cytotoxic T cells, memory T cells, regulatory T cells), NK cells (immature, mature, activated, inhibitory NK cells), and γδ T-cell subsets. Samples were stained with monoclonal antibodies, lysed, washed, and analyzed following standardized protocols to ensure reproducibility and reliability.

Cytokines, including transforming growth factor-β (TGF-β), interleukin-6 (IL-6), interleukin-10 (IL-10), interleukin-12 (IL-12), and interleukin-21 (IL-21), were quantified using intracellular cytokine staining (ICS) via flow cytometry. Peripheral blood mononuclear cells (PBMCs) were stimulated, fixed, permeabilized, and stained with cytokine-specific antibodies according to standardized protocols.

All collected data, including demographic, clinical, biochemical, immune cell subset, and inflammatory cytokine data, were systematically recorded in standardized case report forms. These data provided the basis for evaluating the associations of frailty status with clinical, functional, and immunological characteristics in patients undergoing MHD.

### Frailty evaluation

Frailty status among MHD patients was assessed using the Fried FRAIL scale, a widely validated and recognized tool for evaluating frailty in both clinical and research settings (14, 15). Assessments were conducted through face-to-face interviews by trained physicians and nurses, who completed the questionnaire based on patients’ responses. The FRAIL scale consists of five components, each scored dichotomously as “yes” (1 point) or “no” (0 points), with a total score ranging from 0 to 5. Based on their total FRAIL score, patients were categorized into three frailty groups: Normal (0 points), Pre-Frail (1–2 points), and Frail (3–5 points).

This assessment approach provided a standardized and reliable method for identifying frailty status in MHD patients and enabled structured comparisons of clinical, functional, and immunological parameters across the three frailty subgroups. By stratifying participants into Normal, Pre-Frail, and Frail categories, the study examined the associations between frailty status and a range of clinical and biological outcomes, thereby offering insight into the mechanisms underlying frailty in this vulnerable population.

### Statistical analysis

Continuous variables were expressed as means with standard deviations (SD) for normally distributed data, or as medians with interquartile ranges (IQR) for non-normally distributed data. Categorical variables were presented as frequencies and percentages. Comparisons between the training and validation cohorts were performed using the Student’s t-test for normally distributed continuous variables, the Wilcoxon signed-rank test for non-normally distributed continuous variables, and either the chi-square test or Fisher’s exact test for categorical variables, as appropriate.

All *p* values were two-sided, with a significance level set at 0.05. Statistical analyses were conducted using R software version 4.3.1 (R Foundation for Statistical Computing, Vienna, Austria; http://www.R-project.org).

## Results

### Baseline characteristics

**Figure 1:**
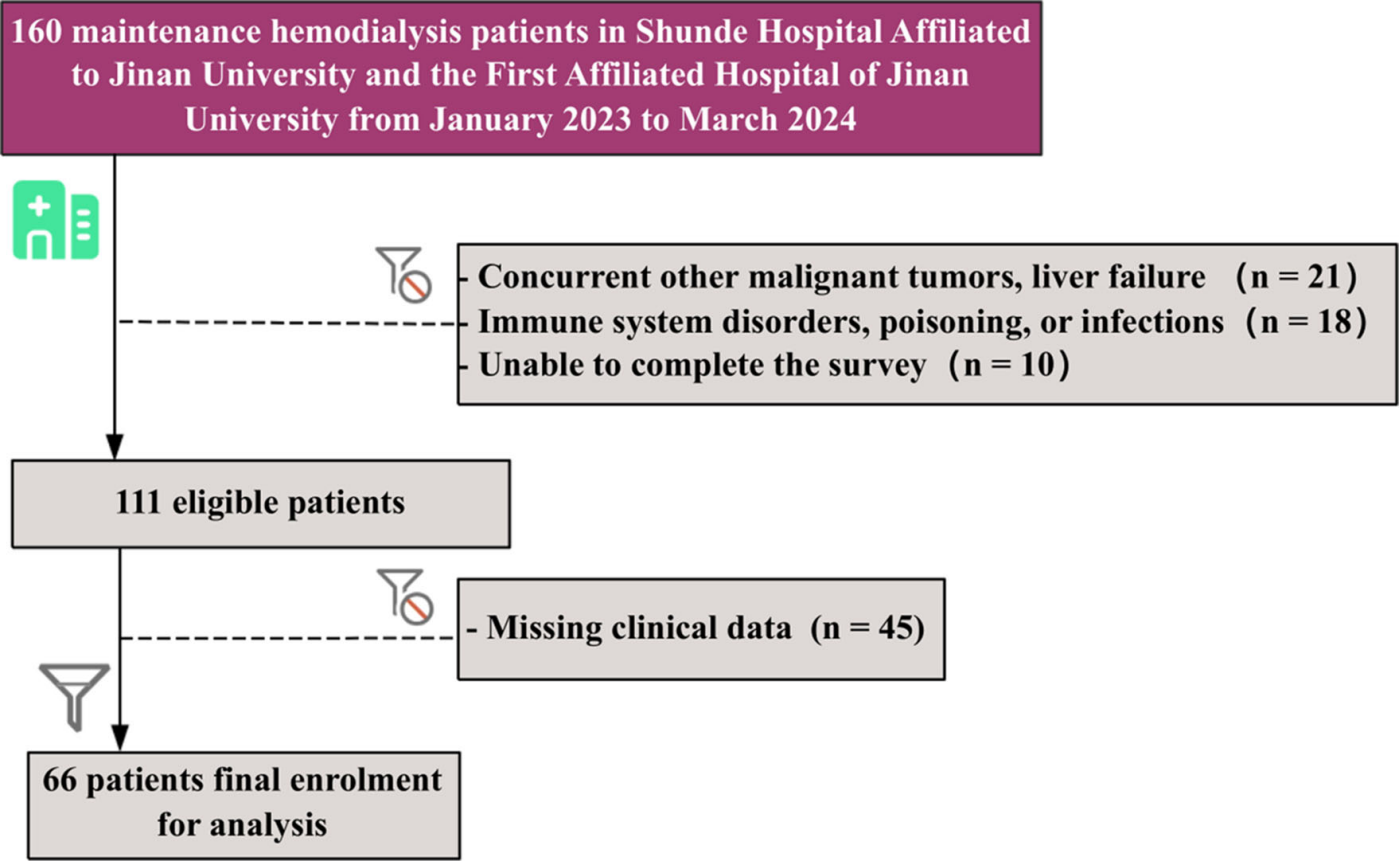
Flowchart depicting the patient enrollment process for the study.

A total of 66 MHD patients were included in the final analysis (**Figure 1**), stratified into three frailty subgroups based on Fried FRAIL scale scores: Normal (n = 15), Pre-Frailty (n = 24), and Frailty (n = 27).

The baseline characteristics are summarized in **Table 1**, with additional details provided in the **Supplementary Table 1**. The mean age of the cohort was 60.52 ± 15.31 years, with patients in the Frailty group significantly older (64.19 ± 14.37 years) than those in the Normal group (49.60 ± 16.44 years, *p* = 0.005). Height was significantly lower in the Pre-Frailty and Frailty groups compared to the Normal group (*p* = 0.041).

**Table 1:** Baseline characteristics with significant differences across frailty groups.

Variable^a^	ALL (N=66)	Normal (N=15)	Pre-Frailty (N=24)	Frailty (N=27)	*p* value^b^
Age, years, (mean ± SD)	60.52 ± 15.31	49.60 ± 16.44	63.21 ± 12.79	64.19 ± 14.37	0.005
Height, cm, (mean ± SD)	161.47 ± 9.53	166.73 ± 6.76	159.08 ± 9.14	160.67 ± 10.32	0.041
Grip Strength, kg, (median [IQR])	18.35 [15.20;24.97]	26.70 [20.90;30.20]	17.65 [12.30;21.10]	18.00 [13.60;22.60]	0.001
History of falls					0.040
No	34 (51.52%)	12 (80.00%)	11 (45.83%)	11 (40.74%)	
Yes	32 (48.48%)	3 (20.00%)	13 (54.17%)	16 (59.26%)	
Physical activity:					<0.001
No	43 (65.15%)	2 (13.33%)	16 (66.67%)	25 (92.59%)	
Yes	23 (34.85%)	13 (86.67%)	8 (33.33%)	2 (7.41%)	
FRAIL Score, (median [IQR])	2.00 [1.00;4.75]	0.00 [0.00;0.00]	1.50 [1.00;2.00]	5.00 [4.00;5.00]	<0.001
SBP, mmHg, (median [IQR])	134.00 [125.00;146.50]	125.00 [123.50;134.00]	133.50 [123.75;142.25]	140.00 [130.00;161.00]	0.047
Ferritin, ug/L, (median [IQR])	108.50 [62.50;214.25]	84.51 [50.30;135.50]	71.05 [46.30;142.50]	207.00 [106.50;297.50]	0.002
iPTH, pg/ml, (median [IQR])	189.75 [104.15;328.20]	303.80 [148.75;500.35]	217.50 [112.47;454.25]	140.50 [86.65;226.95]	0.039
HbA1c (%), (mean ± SD)	6.03 ± 1.59	5.22 ± 1.30	5.93 ± 1.35	6.58 ± 1.75	0.024
Immune Age, years, (mean ± SD)	67.08 ± 13.50	57.53 ± 16.07	69.83 ± 12.02	69.93 ± 11.01	0.006
Effector memory CD4+T cell, (mean ± SD)	45.99 ± 13.40	51.79 ± 10.92	41.18 ± 15.52	47.04 ± 11.40	0.046
Central memory CD8+T cell, (median [IQR])	3.13 [2.04;5.53]	2.12 [1.41;2.48]	3.90 [2.79;5.34]	4.00 [2.20;6.22]	0.013
Th2 (% of Th cells), (median [IQR])	14.30 [10.25;18.68]	10.80 [8.59;15.00]	14.45 [9.92;18.55]	15.90 [11.30;20.95]	0.028
Th1/Th2, (median [IQR])	1.66 [1.21;2.58]	2.25 [1.87;3.04]	1.52 [1.04;2.30]	1.43 [0.99;2.26]	0.013
NK cell (% of lymphocyte), (median [IQR])	9.64 [6.66;16.15]	7.02 [5.15;11.25]	13.70 [9.19;20.00]	8.66 [7.11;12.30]	0.045
Inhibitory γδ2+T cell, (median [IQR])	4.16 [2.89;7.61]	2.83 [2.38;4.75]	3.98 [2.85;4.90]	6.76 [3.71;9.58]	0.005
Conventional killer γδ2+T cell, (median [IQR])	2.22 [1.19;5.07]	1.55 [0.93;1.80]	3.33 [1.99;5.34]	2.50 [0.98;6.25]	0.034

^a^Continuous variables were presented as mean ± SD for normal distribution or median (IQR) for non-normal distribution and categorical variables as Number (%).

^b^*p* values were calculated using the Student’s t-test for normally distributed or Wilcoxon Rank Sum test for non-normally distributed continuous variables and the χ^2^ test for categorical variables.

SD, standard deviation; IQR, interquartile range.

Functional measures differed notably among the groups. Grip strength was highest in the Normal group (26.70 [20.90–30.20] kg) and progressively declined in the Pre-Frailty (17.65 [12.30–21.10] kg) and Frailty groups (18.00 [13.60–22.60] kg, *p* = 0.001). Physical activity levels were markedly lower in the Frailty group, with only 7.41% engaging in regular activity compared to 86.67% in the Normal group (*p* < 0.001). The prevalence of falls was also significantly higher in the Frailty group (59.26%) compared to the Normal group (20.00%, *p* = 0.040).

Biochemical markers showed significant differences, with ferritin levels highest in the Frailty group (207.00 [106.50–297.50] μg/L) compared to the Normal (84.51 [50.30–135.50] μg/L) and Pre-Frailty groups (71.05 [46.30–142.50] μg/L, *p* = 0.002). Hemoglobin A1c (HbA1c) was also elevated in the Frailty group (6.58 ± 1.75%) compared to the Normal group (5.22 ± 1.30%, *p* = 0.024).

Immunological parameters varied significantly across subgroups. Effector memory CD4+ T-cell levels were highest in the Normal group (51.79 ± 10.92%) and decreased in the Pre-Frailty (41.18 ± 15.52%) and Frailty groups (47.04 ± 11.40%, *p* = 0.046). Central memory CD8+ T-cell levels, Th2 percentages, and Th1/Th2 ratios also showed significant differences (*p* < 0.05).

### Comparison across frailty groups

**Figure 2:**
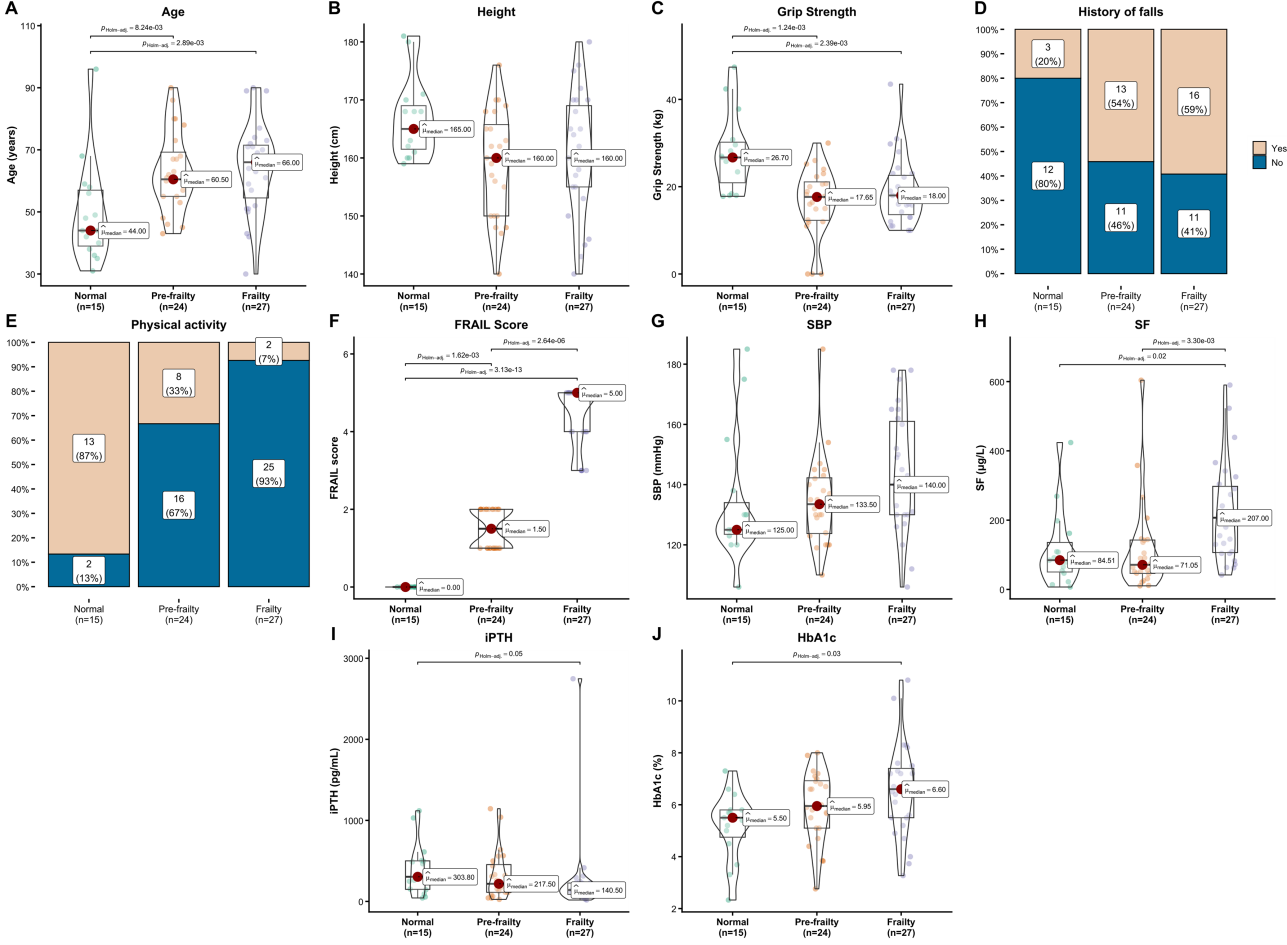
Comparison of clinical and functional characteristics across frailty groups. This figure shows the distributions of clinical and functional variables across three frailty groups: Normal (n = 15), Pre-frailty (n = 24), and Frailty (n = 27). Continuous variables are presented as simplified group-wise distribution plots with overlaid individual data points, allowing visualization of central tendency, data dispersion, and between-group differences. Categorical variables are shown as proportional bar plots with direct count and percentage labels. Only the essential intergroup comparison information is displayed. Panels show: **(A)** Age, **(B)** Height, **(C)** Grip strength, **(D)** History of falls, **(E)** Physical activity, **(F)** FRAIL score, **(G)** systolic blood pressure (SBP), **(H)** ferritin (SF), **(I)** intact parathyroid hormone (iPTH), and **(J)** hemoglobin A1c (HbA1c).

**Figure 3:**
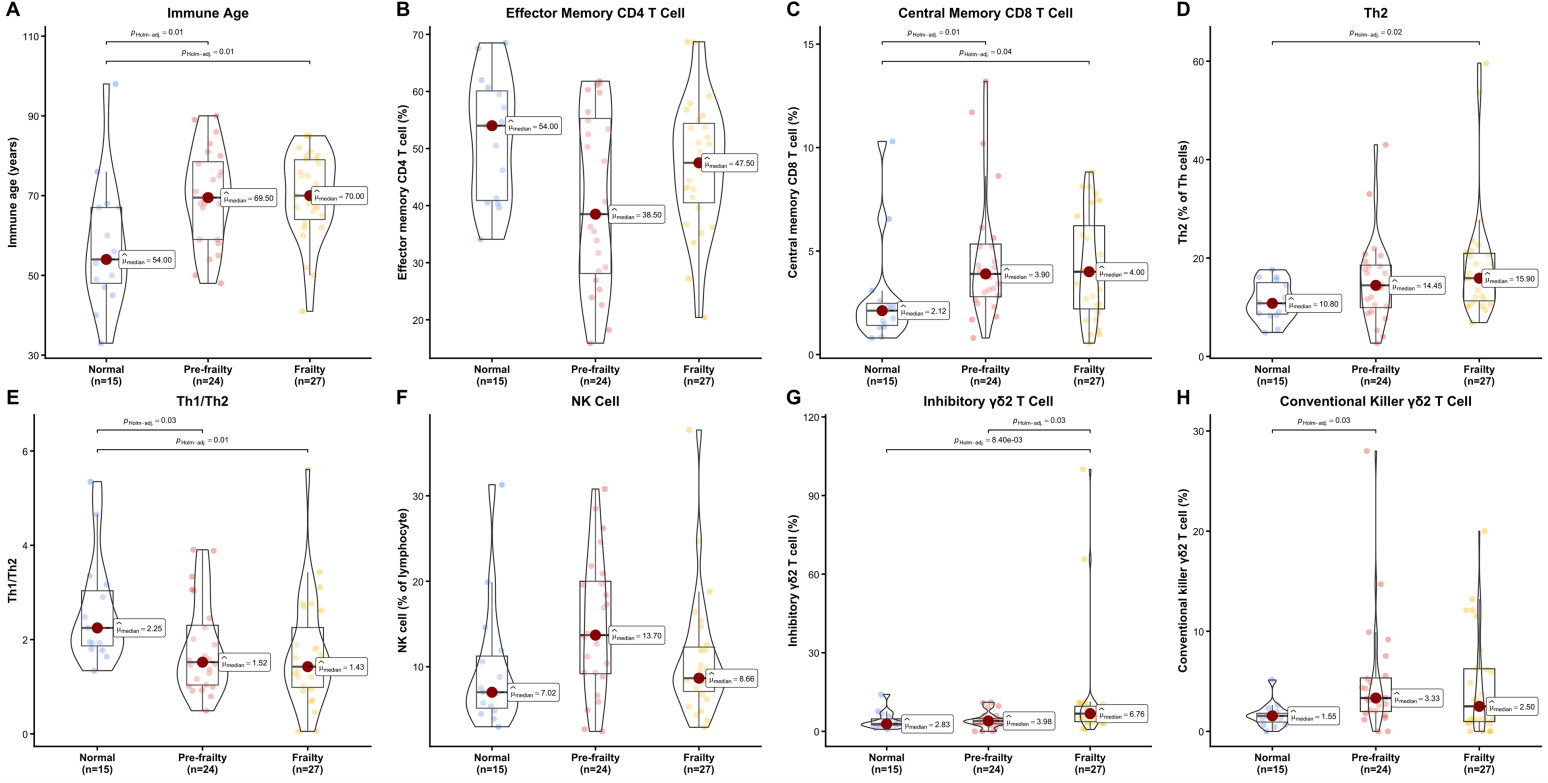
Comparison of immune and inflammatory markers across frailty groups. This figure shows the distributions of immune and inflammatory variables across three frailty groups: Normal (n = 15), Pre-frailty (n = 24), and Frailty (n = 27). Variables are presented as simplified group-wise distribution plots with overlaid individual data points to illustrate central tendency, data dispersion, and between-group differences, while only the essential intergroup comparison information is retained. Panels show: **(A)** Immune age, **(B)** Effector memory CD4 T cells, **(C)** Central memory CD8 T cells, **(D)** Th2 cells (% of Th cells), **(E)** Th1/Th2 ratio, **(F)** NK cells (% of lymphocytes), **(G)** Inhibitory γδ2 T cells, and **(H)** Conventional killer γδ2 T cells.

The clinical, functional, and immunological characteristics of MHD patients varied significantly across frailty subgroups, as illustrated in **Figure 2** and **Figure 3**.

Age and functional parameters showed significant differences across frailty groups. The Frailty group had the highest median age (66.00 years) compared to the Normal (44.00 years) and Pre-Frailty groups (60.50 years, *p* = 0.002, **Figure 2A**). Grip strength also differed notably, with the Frailty group displaying a marked decline (18.00 kg) compared to the Normal (26.70 kg) and Pre-Frailty groups (17.65 kg, *p* < 0.001, **Figure 2C**). Similarly, the prevalence of physical activity was drastically reduced in the Frailty group (7%) compared to the Normal group (87%, *p* < 0.001, **Figure 2E**), and a higher history of falls was observed in the Frailty group (59%) compared to the Normal group (20%, *p* = 0.040, **Figure 2D**).

Biochemical markers, including ferritin, HbA1c, and iPTH, showed significant variability. The Frailty group exhibited higher ferritin levels (207.00 μg/L) compared to the Normal group (84.51 μg/L, *p* = 0.002, **Figure 2H**), and elevated HbA1c levels (6.60%) compared to the Normal group (5.50%, *p* = 0.030, **Figure 2J**).

Immune parameters demonstrated substantial differences across frailty groups, highlighting the systemic immunological changes associated with frailty. Immune age was significantly higher in the Frailty group (70.00 years) than in the Normal group (54.00 years, *p* = 0.010, **Figure 3A**). Effector memory CD4+ T-cell levels were lowest in the Frailty group (47.50%) compared to the Normal group (54.00%, *p* = 0.040, **Figure 3B**). Central memory CD8+ T-cell levels were elevated in the Frailty group (4.00%) compared to the Normal group (2.12%, *p* = 0.010, **Figure 3C**).

Inflammatory markers also varied significantly. Th2 cells (% of Th cells) were highest in the Frailty group (15.90%) compared to the Normal group (10.80%, *p* = 0.030, **Figure 3E**), while the Th1/Th2 ratio decreased with frailty progression (1.43) compared to the Normal group (2.25, *p* = 0.010, **Figure 3F**). NK cell percentages were higher in the Frailty group (13.70%) compared to the Normal group (7.02%, *p* = 0.040, **Figure 3G**), and inhibitory γδ2+ T-cell levels were also elevated (6.76%) compared to the Normal group (2.83%, *p* = 0.010, **Figure 3H**).

### Correlation between immune age and biological age

**Figure 4:**
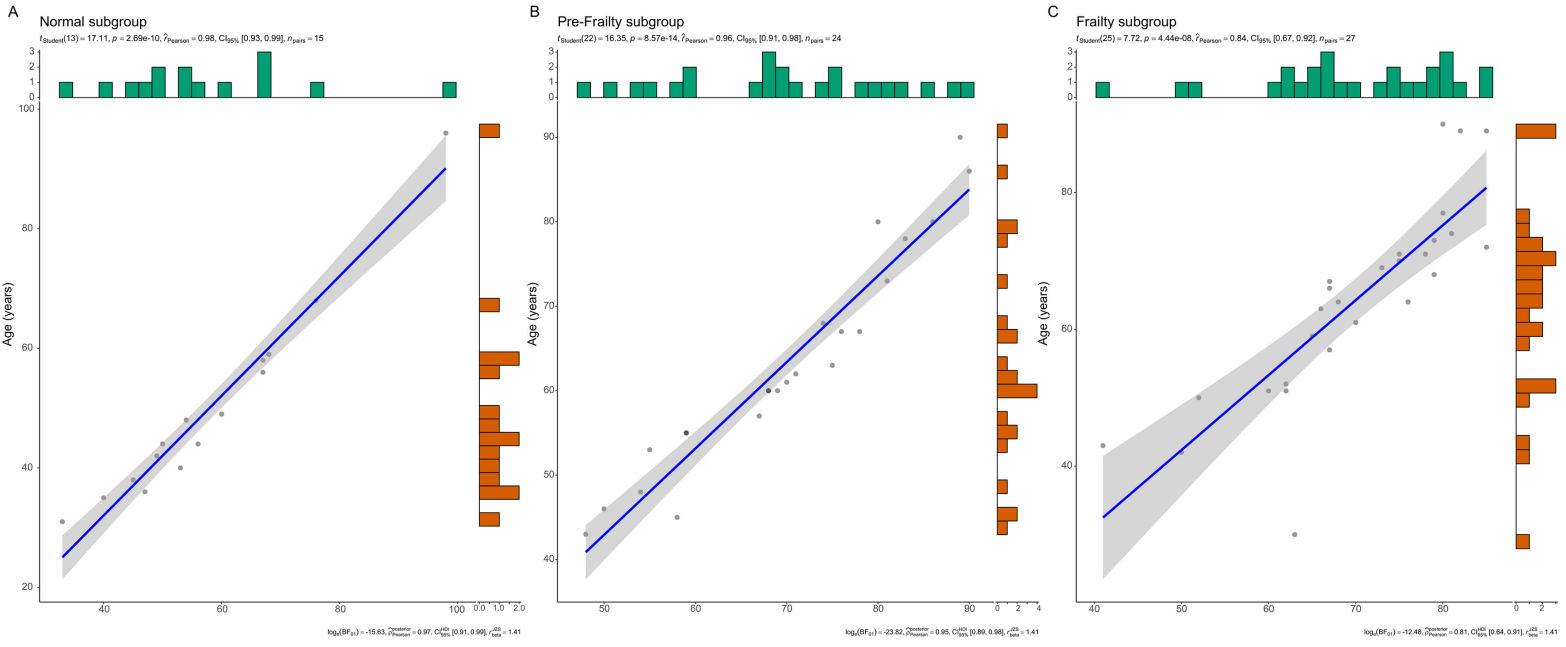
Correlation analysis between age and key variables in frailty subgroups. This figure illustrates the correlation analysis between age and selected key variables across the three frailty subgroups: Normal (n = 15), Pre-Frailty (n = 24), and Frailty (n = 27). Panels **(A–C)** show the results of the correlation analysis for each subgroup.

The correlation between immune age and biological age was analyzed across the three frailty subgroups: Normal, Pre-Frailty, and Frailty, as shown in **Figure 4**.

In the Normal subgroup, immune age and biological age demonstrated a strong positive correlation (r = 0.98, *p* < 0.001), indicating that immune age closely aligned with chronological age in this group (**Figure 4A**). Similarly, in the Pre-Frailty subgroup, the correlation remained strong (r = 0.96, *p* < 0.001), suggesting that immune aging progressed proportionately with biological aging in this group as well (**Figure 4B**). In contrast, the Frailty subgroup exhibited a weaker, albeit still significant, positive correlation (r = 0.84, *p* < 0.001), reflecting a potential dysregulation in immune aging relative to biological age in patients with advanced frailty (**Figure 4C**).

## Discussion

This single-center observational study explored the associations between frailty status and clinical, functional, and immunological characteristics in patients undergoing MHD, showing that frailty in this population is a multidimensional condition involving not only physical decline but also measurable immune alterations. By integrating clinical, functional, biochemical, inflammatory, and immune cell subset data, the study provided a more comprehensive characterization of heterogeneity across frailty subgroups. In this context, the main contribution of our study lies in highlighting the potential value of immunological profiling and immune age assessment as complementary tools for frailty evaluation in MHD patients.

Our findings showed that frailty was associated with older age, reduced grip strength, lower physical activity, and a higher prevalence of falls. These observations are broadly consistent with previous studies and confirm the expected functional vulnerability of frail MHD patients (16, 17). The significant reduction in physical activity in the Frailty group further emphasizes the importance of preserving mobility and promoting structured exercise to delay frailty progression (18, 19), while the increased history of falls supports the need for targeted fall-prevention strategies in this high-risk population (20). However, the originality of the present study lies not primarily in these expected clinical findings, but in the observation that frailty subgroups also differed in immune-related characteristics, suggesting that frailty in MHD patients may reflect a broader process of immune remodeling and dysregulation rather than physical impairment alone (21). These immune abnormalities may also have clinical implications, as altered immune cell subsets and immune-inflammatory imbalance could reflect underlying immunosenescence and chronic immune dysregulation, potentially contributing to reduced physiological reserve and greater vulnerability to adverse outcomes (22). In addition, the biochemical abnormalities observed in the frailty group, particularly elevated ferritin and HbA1c, may be clinically meaningful. Higher ferritin levels may reflect chronic inflammation and disordered iron metabolism, whereas elevated HbA1c may indicate poorer glycemic control and metabolic dysregulation; both processes have been associated in previous studies with frailty progression and unfavorable prognosis.

Importantly, beyond these expected functional differences, our study further identified significant variations in immune cell subsets and immune-inflammatory balance across frailty stages. Ferritin and HbA1c were significantly elevated in the Frailty group, whereas iPTH levels were significantly lower. Elevated ferritin may reflect chronic inflammation and disordered iron metabolism, both of which have previously been linked to frailty and adverse clinical outcomes in dialysis patients (23). Likewise, higher HbA1c levels may indicate poorer glycemic control and metabolic dysregulation, which may contribute to frailty progression (24, 25). In contrast, the lower iPTH levels observed in frail patients may suggest altered bone–mineral metabolism regulation or a relatively low-bone-turnover state in the setting of advanced physiological vulnerability, although this finding should be interpreted cautiously. Taken together, these biochemical abnormalities may provide clinically relevant information and may complement functional assessment in identifying frailty-related vulnerability in MHD patients.

Immune parameters, including effector memory CD4^+^ T cells, central memory CD8^+^ T cells, and inflammatory markers such as Th2%, Th1/Th2 ratio, NK cells, and inhibitory γδ2^+^ T cells, also varied significantly across frailty groups. The observed decrease in effector memory CD4^+^ T cells and increase in central memory CD8^+^ T cells in the Frailty group may reflect immune senescence and impaired adaptive immune responsiveness (26). In parallel, the increased Th2 proportion and decreased Th1/Th2 ratio suggest an imbalance in immune regulation, possibly indicating chronic immune activation, exhaustion, or a shift in immune homeostasis (27). These findings s are consistent with emerging evidence linking immunosenescence and chronic low-grade inflammation to frailty progression in dialysis patients (28, 29). From a clinical perspective, such immune abnormalities may have potential value for frailty stratification, risk monitoring, and the identification of patients who may benefit from closer follow-up or more individualized supportive interventions.

The correlation analysis between immune age and biological age revealed distinct patterns across the frailty subgroups. In the Normal and Pre-Frailty groups, immune age closely aligned with biological age, reflecting synchronized immune and physiological aging processes. In contrast, this relationship was weakened in the Frailty group, which may indicate increasing immune dysregulation and a partial decoupling of immune aging from chronological or biological aging in more vulnerable patients (30). This finding further supports the potential value of immune age as a complementary biomarker in frailty assessment. From a clinical perspective, immune age may help refine risk stratification and monitoring by identifying patients in whom biological vulnerability is accompanied by more pronounced immune alteration. However, given the observational design, this finding should be interpreted as associative rather than causal and warrants confirmation in future longitudinal studies.

## Limitations

The strengths of this study include the multidimensional assessment of frailty, combining clinical, functional, and immunological parameters, and the use of validated tools such as the FRAIL scale and flow cytometry-based immune profiling. However, several limitations should be acknowledged. First, the single-center observational design precludes causal inference, and the relatively small sample size may limit the generalizability of the findings. Second, although some anthropometric and dialysis-related variables, such as dry weight, BMI, and dialysis vintage, were available, other clinically relevant factors, including the primary disease of ESKD and vascular access type, were not systematically collected and therefore could not be evaluated for their potential associations with frailty status or inflammatory markers. In addition, potential confounders, such as nutritional status, medication use, and treatment-related factors including erythropoietin use, were not fully accounted for in the analysis.

Future longitudinal studies with larger sample sizes are warranted to clarify the causal relationships between frailty, immune dysfunction, and clinical outcomes in MHD patients. More comprehensive data collection, including dialysis-related characteristics, treatment-related factors, and other potential confounders, will be important for improving risk stratification and mechanistic understanding. Investigating targeted interventions, such as exercise programs, anti-inflammatory therapies, and nutritional support, across different frailty stages may also provide valuable insights into frailty prevention and management in this vulnerable population.

## Conclusions

This study identified significant clinical, functional, and immunological differences across frailty subgroups in MHD patients and found important associations between immune age and biological age. The findings support a multidimensional approach to frailty assessment and suggest that immune-related markers may aid early identification and risk stratification. However, given the observational design, these results should be interpreted as associative rather than causal and require confirmation in future longitudinal studies.

## Data Availability

The original contributions presented in the study are included in the article/**Supplementary Material**. Further inquiries can be directed to the corresponding author.
